# Missing microbiomes: global underrepresentation restricts who research will benefit

**DOI:** 10.1172/JCI183884

**Published:** 2024-07-15

**Authors:** Kevin S. Blake

**Affiliations:** Washington University School of Medicine in St. Louis, St. Louis, Missouri, USA.

As you read this, trillions of microbes are growing on your face, between your teeth, and deep within your gut. To them your body is like a rainforest: rich in nutrients, full of nooks and crannies to nest in, and overall teeming with life. Some species are just transient visitors, carried along by the food you eat or the air you breathe. Others have been with you since the day you were born.

The communities of microbes living on and within the human body are called the microbiome. The past 20 years of microbiome research has produced a major shift in our understanding of human health. One key finding is that the microbiome is not merely a collection of microscopic passengers, clinging to our bodies like barnacles on a ship. Instead, it’s so systematically integrated with human biology it’s better understood as a new organ system (1). Unhealthy microbiome states have been implicated in a growing number of diseases, including obesity, chronic inflammation, and cancers. But an exciting prospect is that this is an organ that can be repaired without surgery. Noninvasive therapies, such as the ingestion of probiotics or prebiotics, could be as effective at treating diseased microbiomes as open-heart surgeries are for diseased cardiovascular systems.

However, a deep inequity undermining this promise is that microbiome research to date has not captured most of the world’s population. Though North America and Europe represent only 14% of the global population, 71% of studied microbiome samples are from Western countries (2). The United States alone represents just 4% of the population but 40% of microbiome samples. In contrast, countries from Central and Southern Asia make up 26% of the global population but just 2% of samples. The 47 “least developed countries” according to the United Nations account for 14% of the world’s population but just 3% of microbiome samples; 29 of these countries have zero samples.

Nationality has no basis in biology. It’s a social category not a biological one. Differences in health outcomes between countries are not due to inherent differences between peoples, but the influence of many factors, including healthcare access, diet, lifestyle, medication use, and environmental exposures (3, 4). Thousands of studies have examined how these factors affect the microbiomes of people from a handful of wealthy, industrialized countries, but we know next to nothing about microbiomes from low- and middle-income countries even though these represent over 80% of the global population (5, 6).

A primary goal of microbiome research is to establish what constitutes a “healthy” microbiome. This can then be used as a baseline to study how diseased microbiomes deviate from healthy ones and as a guidepost when steering them back to health. But just as the forests of North America and savannas of Africa can both be healthy ecosystems while being substantially different, microbiomes from different countries can also be different while still being healthy. Without an understanding of these differences it’s likely that the promised wave of microbiome-based therapeutics will not work outside of the countries in which they were developed (7).

Research focused solely on Western microbiomes will miss functions essential to the health of other populations. For example, gut microbes help us digest and absorb nutrients from plant fibers by producing enzymes our bodies can’t make. Digesting seaweed requires different enzymes than land plants, and since gut microbes in North American populations don’t have these seaweed-specific enzymes those people can’t digest it. However, these enzymes are produced in the microbiomes of Japanese populations, where seaweed is an important part of their daily diet (8).

Furthermore, studying underrepresented populations can improve understanding about all microbiomes. For example, it had long been assumed that Western populations contain antibiotic-resistant bacteria in their microbiomes because of frequent antibiotic use throughout life. However, it was discovered that the microbiomes of uncontacted Yanomami Amerindians from Venezuela, who had no known exposures to commercial antibiotics, also contain resistant bacteria (9). This suggests that antibiotic resistance is a general feature of the human microbiome overall, which is important information when developing strategies to address its spread.

Global disparities in scientific research go far beyond the microbiome field, and true solutions will require sociopolitical changes that biologists cannot address alone. But increased representation in microbiome studies is an important step researchers can begin today. Encouragingly, this is already beginning to happen for countries in Eastern and Southeastern Asia; however, Africa and Western Asia remain substantially underrepresented (2). Additionally, local researchers should be included at every stage of the research pipeline to ensure that the problems and priorities of their countries are being addressed (10, 11). The decreasing cost and increasing portability of DNA sequencing technologies makes building bioinformatics infrastructure in low-resource settings increasingly feasible, and this should be prioritized as well. These efforts aren’t trivial, but a more global perspective will improve microbiome science overall and ensure its benefits extend to everyone.

## Version 1 07/15/2024

Electronic publication
